# Supplementary material for: MEME-LaB: motif analysis in clusters
Source: Bioinformatics. 2013 May 14;29(13):1696–7. doi: 10.1093/bioinformatics/btt248 (PMC3694638; doi:10.1093/bioinformatics/btt248)
Supplement: Supplementary Data [file supp_btt248_Supplementary_Data.zip › Supplementary_Data/Results_Files/most_similar_logos/S-000115.html]

Motif S-000115


Motif S-000115

|  |  |  |
| --- | --- | --- |
| Natural name: | P$SEF3MOTIFGM | More information from PLACE |
| Length: | 6 |  |
| Pathway: | Unknown |  |
